# Anesthetic Management for a Patient with Rosai-Dorfman Disease, Cowden Syndrome, and Lhermitte-Duclos Disease: An Extremely Rare Disease Combination

**DOI:** 10.7759/cureus.44318

**Published:** 2023-08-29

**Authors:** Emily E Weeden, Amreesh Mahil, Jeffrey Huang

**Affiliations:** 1 Anesthesiology Department, Moffitt Cancer Center, Tampa, USA; 2 Morsani College of Medicine, University of South Florida, Tampa, USA

**Keywords:** airway disorders, tracheal hamartoma, lhermitte-duclos disease, cowden syndrome, rosai-dorfman disease

## Abstract

Rosai-Dorfman disease (RDD) is a rare condition that causes massive lymphadenopathy, most commonly in the cervical area. Cowden syndrome (CS) causes hamartomas in the skin and mucosa and predisposes individuals to various malignancies. Lhermitte-Duclos disease (LDD), or dysplastic cerebellar gangliocytoma, is often associated with CS. A 41-year-old female with all three conditions presented with abnormal uterine bleeding and endometrial intraepithelial neoplasia (EIN). Precautions should be considered when evaluating patients with RDD and CS preoperatively and during airway management owing to the potential for multisystem involvement, anatomical distortion, and difficult airways. The likelihood of having all three conditions is extremely rare.

## Introduction

Rosai-Dorfman disease (RDD) is a rare disease of unknown cause that usually presents in adolescence and is characterized by massive cervical lymphadenopathy. Other nodal sites such as supraclavicular and submandibular lymph nodes can also be involved [1]. Extranodal disease is commonly seen in the head and neck, including within the nasal cavity, salivary glands, oral cavity, pharynx, tonsils, and trachea [2]. As a result, RDD can cause anatomical distortion and possible extension of soft tissue into the trachea [1].

Cowden syndrome (CS) is the result of autosomal dominant inherited mutations in the tumor suppressor gene PTEN, which leads to unregulated cell growth and hamartoma formation [3-4]. CS is marked by pathognomonic mucocutaneous lesions, such as facial trichilemmomas and papular or verrucous oral lesions of the lips, tongue, gingiva, buccal mucosa, palate, tonsillar fossa, larynx, and vocal cords [3-4]. Unregulated cell growth in CS in particular leads to hamartoma formation in the central nervous system, breast, thyroid, and bones, and predisposes individuals toward developing certain malignancies, most commonly thyroid, breast, and endometrial cancer [5]. One manifestation of CS hamartoma formation in the CNS is Lhermitte-Duclos disease (LDD), also known as dysplastic cerebellar gangliocytoma [5]. LDD can cause mass effect, hydrocephalus, and even brain herniation and death [5].

The prevalence of RDD and CS are both estimated to be 1:200,000, whereas the prevalence of LDD is estimated to be less than 1:1,000,000 [6-8]. However, to our knowledge, there are no reported cases describing individuals expressing all three diseases simultaneously. Thus, the patient we discuss here presented with the possibility of difficult intubation and anesthetic management due to the multisystem involvement of RDD and CS, ultimately warranting preparedness for the anesthesia provider intraoperatively.

## Case presentation

We present a case report involving a 41-year-old female who had Cowden syndrome (CS) due to a germline PTEN mutation, as well as other conditions including Lhermitte-Duclos disease (LDD) and Rosai-Dorfman disease (RDD). The patient underwent an examination under anesthesia, hysteroscopy, dilation and curettage, and polypectomy. The diagnoses of CS, LDD, and RDD were established at a different cancer hospital through clinical presentations, radiographic examinations, and biopsies, encompassing breast, thyroid, skin, endometrial, and bone biopsies, along with genetic testing. In addition to these conditions, the patient's medical history included asthma, gastroesophageal reflux disease (GERD), hyperlipidemia, hypothyroidism, obesity, and a left parotid mass. Notably, the patient had a history of a bone lesion in the right lower extremity characterized by lymphohistiocytic proliferation, gangliocytoma, and multinodular thyroid.

The patient's surgical history encompassed multiple left breast biopsies, a right oophorectomy, a bone biopsy, thyroid biopsy, and a skin biopsy. Over a span of two years before the procedure, the patient had undergone evaluation by various gynecologic providers on multiple occasions. These evaluations revealed endometrial intraepithelial neoplasia (EIN), a potential precursor to endometrial cancer, leading to a referral for treatment to address abnormal uterine bleeding (AUB) and associated symptoms, while still desiring future fertility. The patient was taking levothyroxine and ezetimibe-simvastatin as medications and was allergic to sulfamethoxazole-trimethoprim and ciprofloxacin.

Preoperative assessments of the patient included an electrocardiogram that showed normal [E1] sinus rhythm and an echocardiogram performed two years prior and a carotid ultrasound. The echocardiogram demonstrated normal left ventricular size and systolic function, with a left ventricular ejection fraction of 50-55%. Grade 1 left ventricular diastolic dysfunction characterized by impaired relaxation and normal filling pressures was also present. Finally, trace mitral and tricuspid valve regurgitation were also noted. The carotid ultrasound demonstrated heterogenous plaquing with less than 40% stenosis of the bilateral internal and external carotid arteries, alongside normal flow in the bilateral vertebral arteries. Preop CBC and CMP were within normal limits with the following values: hemoglobin 13.0, platelets 275. CMP revealed Na 140, K 4.6, Cl 106, HCO3 29, BUN 9, Cr 0.6, and glucose 95. The urine pregnancy test was negative. Blood type was A+ with a negative antibody screen. Regarding physical measurements, the patient’s height, weight, and BMI were recorded as 164 cm, 113 kg, and 43.5 respectively. During the examination, the patient exhibited a short neck, a mouth opening of 5 cm, and thyromental distance of greater than 6 cm, with a normal neck range of motion. The patient’s airway was classified as Mallampati III, indicating potential airway difficulty.

Given the patient’s history and physical attributes, the anesthesia providers involved anticipated a potentially challenging airway. To address this concern, preparations were made with a difficult airway cart, Glidescope (Verathon Medical, Bothell, USA), and bronchoscope immediately available. Experienced anesthesia personnel were also on standby ready for intervention. Finally, the anesthesia team reviewed the pertinent literature and discussed possible anesthetic plans prior to the case.

Preoperatively, the patient was normotensive and hemodynamically stable, with the following vitals: blood pressure was 134/70, HR 100, O2 sat 100%, and respiratory rate 12. The patient was then given 2 mg of midazolam preoperatively for anxiolysis. Following preoxygenation with 100% O2, general anesthesia was induced with fentanyl (100 mcg), lidocaine (80 mg), propofol (200 mg), and rocuronium (50 mg). A paralytic was administered after confirmation that the patient was easy to mask and ventilate. Video laryngoscopy with a size 3 laryngoscope was used to assist intubation, during which a size 7.0 mm endotracheal tube was placed without difficulty or trauma. Auscultation revealed bilateral breath sounds. Anesthesia was maintained with inhaled desflurane at a minimal alveolar concentration (MAC) of 0.8-1.0. Air and oxygen were also administered. Intraoperatively, hemodynamic and respiratory statuses remained stable, oxygen saturation (SaO2) remained 100%, and temperature was maintained above 36.5°C. The patient’s EKG consistently demonstrated normal sinus rhythm throughout the case. Extubation occurred at the end of the surgery, and the patient was transferred to the post-anesthetic care unit (PACU). The patient’s postoperative course was uneventful.

## Discussion

Due to the rare prevalence of CS, LDD, and RDD, the likelihood of an individual expressing all three diseases simultaneously is incredibly rare. CS is seen in those with a PTEN mutation, of which approximately half are familial and half are sporadic [5]. PTEN, a tumor suppressor gene, normally limits the phosphorylation of PIP3. When PTEN is mutated and therefore defective at limiting the phosphorylation of PIP3, PIP3 can activate PDK1. PDK 1 in turn phosphorylates Akt, and phosphorylated Akt drives cellular processes including cell growth. This process is what occurs in autosomal dominantly inherited cases of CS and leads to unregulated cell and tumor growth, leading to the hamartoma formation of CS [5]. The manifestations of CS present during adolescence, whereas LDD is a slow-growing tumor that often does not develop until the third or fourth decade of life with slow progressive cerebellar symptoms and symptoms of increased intracranial pressure [5,8-10]. LDD is typically sporadic, however, when associated with CS it is of autosomal dominant inheritance due to PTEN mutations [8]. [E1] RDD is more commonly seen in children and young adults [11]. Interestingly, the pathogenesis of RDD is not well defined. Some suspect that RDD is autoimmune in nature or a reaction to an unknown infectious agent [1-2].

CS is diagnosed via pathognomonic, major, and minor criteria, some of which include select mucocutaneous lesions, macrocephaly, LDD, and breast, endometrial, thyroid, and renal cell carcinoma [12]. Of note, LDD is considered a pathognomonic diagnostic criterion of CS [5]. Due to the slow progressive development of LDD, patients commonly remain asymptomatic for many years [8]. LDD is also commonly found incidentally [8]. In addition to signs of increased ICP and cerebellar dysfunction, LDD can present with macroglossia, vascular malformations, and leontiasis ossea, a severe form of bone remodeling that causes great enlargement of the facial bones, especially the maxilla and mandible [8-9].

RDD is diagnosed based on a constellation of findings. On an exam, one will note bilateral, massive, painless cervical lymphadenopathy [11]. Extranodal involvement of RDD can be seen in the oral cavity, leading to soft and hard palate nodules, gingival and oral mucosa swelling, macroglossia, and enlarged tonsils [11]. Less-frequent extranodal sites of involvement include the salivary and parotid glands, larynx, pharynx, thymus, and thyroid gland causing symptoms related to mass effect [11]. Due to the massive lymphadenopathy posed by RDD, there is a possibility for difficult mask ventilation [1]. Some have suggested the role of awake intubation in those with RDD in order to limit risk of respiratory compromise [1]. RDD can even precipitate intratracheal extension of soft tissue mass, making passage of an endotracheal tube difficult [1]. Ultimately, the disease manifestations of RDD can cause distortion of airway anatomy and consequently pose difficulties during airway management.

Similarly, papillomatous formations secondary to CS can go undetected during routine preoperative evaluation and obscure anatomic landmarks, such as the epiglottis [4]. Thus, upon attempt at intubation, it is possible for these friable mucocutaneous lesions to cause bleeding amidst intubation, threatening the visualization of the airway [E2] [4]. Even with proper visualization of the vocal cords prior to intubation, there have been documented cases of CS-related mucocutaneous lesions causing bleeding mid-intubation, requiring the use of a fiberoptic bronchoscope to facilitate the placement of the endotracheal tube (ETT) [13]. Nevertheless, difficulties with intubation have also been documented in patients with CS while using fiberoptic intubation [4]. Moreover, the use of a Glidescope [E3] and the patient history of prior uneventful intubations have not ruled out the possibility of encountering future complications during anesthesia [13]. However, some providers have noted success when maintaining visualization via the Glidescope and using a fiberoptic bronchoscope as a stylet for the ETT [13]. The use of Glidescope and fiberoptic bronchoscope had been used for successful intubation of a patient with CS [13]. Fiberoptic-guided oral tracheal intubation has also been noted to be successful in a case of RDD with gross bilateral cervical, supraclavicular, and submandibular lymphadenopathy, a neck circumference of 44 cm, and restricted mouth opening and neck extension [1]. Soft tissue mass was also noted to be in the nasopharynx, oropharynx, nasal cavities, pterygoid fossa, and soft palate [1].

Ultimately, CS and RDD can cause distortion of the airway and lead to unexpectedly difficult airway management both in known and unknown cases. Because of this, anesthesiologists should have a high level of suspicion for the possibility of difficult airway management prior to starting a case. While it is imperative to perform a thorough preoperative evaluation of all patients, it is especially necessary in those with known disease in order to limit surprise difficult airway management. Even if someone has undergone anesthesia previously without difficulty or complication, this does not prevent the possibility of future difficult anesthetic management owing to the evolving nature of RDD and CS. One’s pathology may change over time and lead to a future unexpected difficult intubation. Additionally, as a result of CS and RDD’s potential to evolve, extensive preoperative evaluation is warranted in known cases prior to every need for anesthesia.

Thus, when assessing patients preoperatively, noting the location and friability of mucocutaneous lesions in CS is important and may change anesthetic management [14]. One may also note the presence of macrocephaly, hypoplastic maxilla, high-arched palate, hypoplastic mandible, or thyroid tumors in patients with CS, which can encumber intubation [14]. Some have even proposed the utility of airway imaging preoperatively via MRI or CT in order to evaluate one’s pathology location and extent [13]. Depending on the extent of one’s disease involvement, providers may also consider avoiding the use of nasogastric tubes and nasal intubation [14]. Some have also questioned the utility of supraglottic airways in cases of CS due to the friable nature of the papillomatous lesions [14]. Because RDD can lead to intratracheal extension of soft tissue mass, and CS can cause mucocutaneous lesions in the oral cavity and pharynx, a preoperative sleep study has also been suggested to assess possible difficulty with mask ventilation and airway obstruction [1,13].

In addition, patients with either RDD or CS may have undergone local resection, radiotherapy, or chemotherapy [11,14]. Radiotherapy in particular to the airway or nearby structures puts a patient at risk for difficult airway management. Thus, a thorough preoperative history is equally as important as physical evaluation and imaging. Additionally, if a known case is symptomatic secondary to manifestations of either RDD or CS, and symptomatic in such a way that poses a risk to undergoing anesthesia, one may even consider recommending treatment preoperatively in order to best optimize a patient for surgery [11]. Most importantly, due to the manifestations of RDD and CS, anesthesia providers taking care of patients with RDD or CS may encounter difficult mask fit, bag-mask ventilation, and intubation.

**Figure 1:**
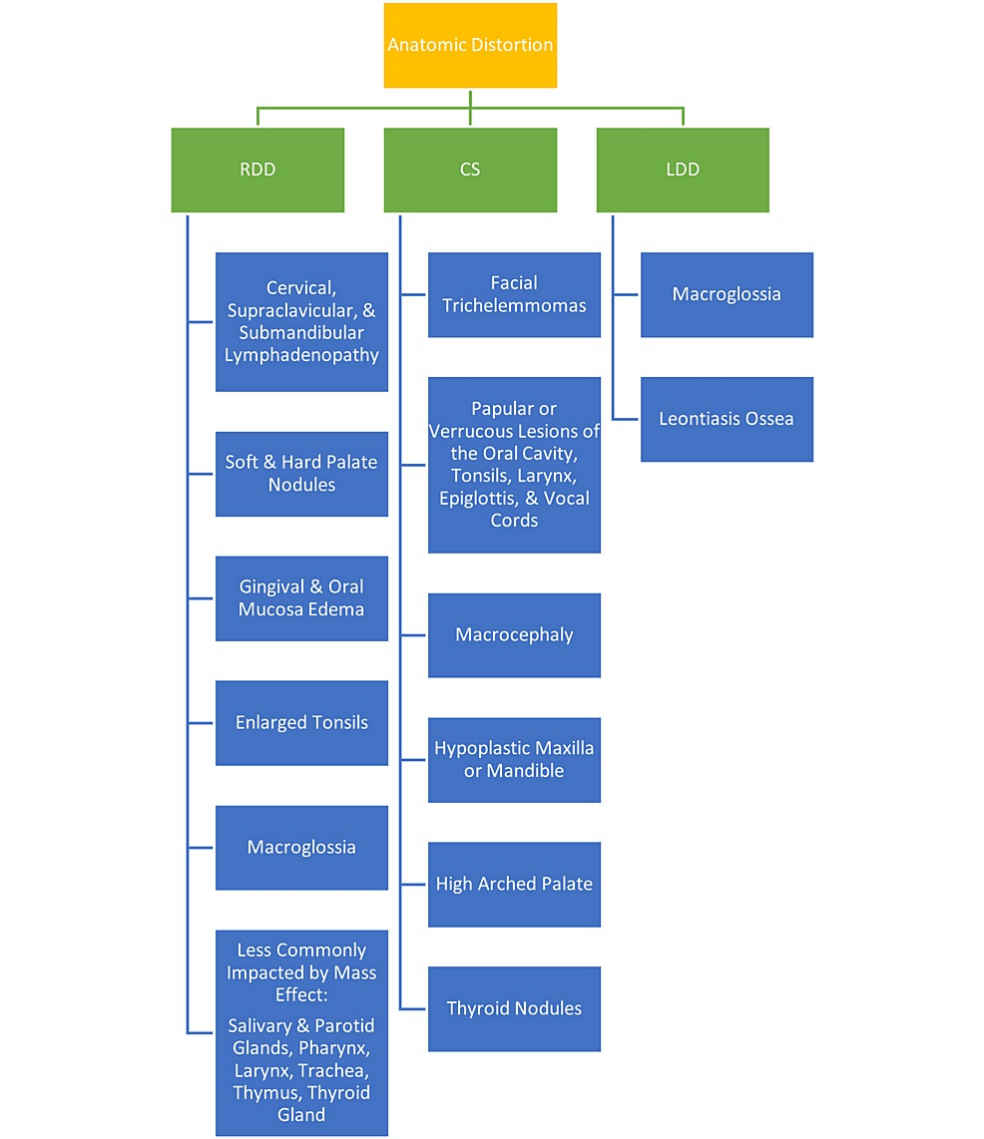
Anatomic Overview of RDD, CS, and LDD Sources: [1,3,4,8,9,11,12,14]

Due to the potential for difficult airways in patients with RDD and CS, extra preparedness is key (Figure 1). As a result, appropriate equipment, including a difficult airway cart and additional assistance, should be readily available when managing a patient with RDD or CS. Regarding the use of a Glidescope versus a fiberoptic bronchoscope either with or without visualization via the Glidescope in patients with RDD or CS, further investigation is required. The Glidescope provides the user with greater visualization of the patient’s anatomy; however, the use of such still does not guarantee the avoidance of trauma. Similarly, while the fiberoptic bronchoscope is easily maneuverable and may be used as a stylet to railroad the ETT past the vocal cords, one may encounter difficulty in doing so, which has the potential for trauma to nearby structures. It is also important to recognize that due to the difficulty in identifying undiagnosed cases via standard preoperative assessment, CS and RDD will continue to pose surprise and difficult airway management.

Regarding the case presented here, extra preparedness was taken in order to limit injury and prevent respiratory compromise. For example, key resources such as a difficult airway cart, multiple strategies to assist with intubation, and experienced anesthesia providers were all readily available during the case. Prior to the case, a review of relevant literature and patient history prompted providers to choose video laryngoscopy to assist with intubation. This was done in order to provide the greatest visualization while limiting the potential for injury. Moreover, the use of fentanyl was also limited to induction in order to prevent possible respiratory depression after extubation, especially considering the patient’s potential for a difficult airway. Lastly, the patient was found to be easily mask-ventilated prior to the administration of the paralytic and was successfully intubated without any adverse events. While there are known cases of CS, LDD, and RDD in isolation, to our knowledge, this is the first case of a single patient expressing all three diseases. This case presents the anesthetic management and considerations for a case with all three conditions simultaneously.

## Conclusions

Few are likely to encounter patients with CS, LDD, and RDD simultaneously. However, when providing anesthesia to patients with CS or RDD, it is imperative for providers to thoroughly evaluate the patient's airway anatomy prior to any procedure and take extra precautionary measures when administering anesthesia due to the possibility of unexpected difficult airway management. In our case here, a 41-year-old female with known CS, LDD, and RDD presented for an exam under anesthesia, hysteroscopy, dilation and curettage, and polypectomy. Due to the patient’s history, she warranted thorough preoperative evaluation and assessment of the degree of organ involvement with special emphasis placed on airway management. Ultimately, with preparedness for the challenges of airway and anesthetic management, our patient had an uncomplicated anesthetic course.
